# A modified particle swarm optimization rat search algorithm and its engineering application

**DOI:** 10.1371/journal.pone.0296800

**Published:** 2024-03-28

**Authors:** Manish Kumar Singla, Jyoti Gupta, Mohammed H. Alsharif, Mun-Kyeom Kim

**Affiliations:** 1 Department of Interdisciplinary Courses in Engineering, Chitkara University Institute of Engineering & Technology, Chitkara University, Rajpura, Punjab, India; 2 Applied Science Research Center, Applied Science Private University, Amman, Jordan; 3 School of Engineering and Technology, K. R. Mangalam University, Haryana, Gurgaon, India; 4 Department of Electrical Engineering, College of Electronics and Information Engineering, Sejong University, Seoul, Korea; 5 School of Energy System Engineering, Chung-Ang University, Dongjak-gu, Seoul, Republic of Korea; Vellore Institute of Technology, INDIA

## Abstract

Solar energy generation requires photovoltaic (PV) systems to be optimised, regulated, and simulated with efficiency. The performance of PV systems is greatly impacted by the fluctuation and occasionally restricted accessibility of model parameters, which makes it difficult to identify these characteristics over time. To extract the features of solar modules and build highly accurate models for PV system modelling, control, and optimisation, current-voltage data collecting is essential. To overcome these difficulties, the modified particle swarm optimization rat search algorithm is presented in this manuscript. The modified rat search algorithm is incorporated to increase the PSO algorithm’s accuracy and efficiency, which leads to better outcomes. The RSA mechanism increases both the population’s diversity and the quality of exploration. For triple diode model of both monocrystalline and polycrystalline, PSORSA has showed exceptional performance in comparison to other algorithm i.e. RMSE for monocrystalline is 3.21E-11 and for polycrystalline is 1.86E-11. Similar performance can be observed from the PSORSA for four diode model i.e. RMSE for monocrystalline is 4.14E-09 and for polycrystalline is 4.72E-09. The findings show that PSORSA outperforms the most advanced techniques in terms of output, accuracy, and dependability. As a result, PSORSA proves to be a trustworthy instrument for assessing solar cell and PV module data.

## 1. Introduction

### 1.1 Overview

Increasing attention has been paid in recent years to the importance of simulating accurately solar PV cells. Two steps are involved in solar cell modelling, namely formulation of mathematical expressions and estimation of cell parameters. Several researchers have reported on a mathematical model derived from the data [1, 2]. Various models of PV cells are commonly used, such as the single diode (SD) model [3], the double diode (DD) model [4], and the three diode (TD) model [5]. In order to use these models to calculate PV panel parameters, specific PV panel parameters need to be estimated. These parameters include saturation current (Id), series resistance (Rse), shunt resistance (Rsh), ideality factor (a), and photocurrent (I). In order to estimate the parameters of the SD, DD, or TD model, depending on the criteria chosen, there is a need to estimate five, seven, or nine parameters, respectively. It is crucial that the optimal values for the aforementioned parameters are determined in order to ensure that the chosen PV model produces outputs that are comparable to those of physical solar cells [6, 7].

### 1.2 Techniques

Solar cells’ parameters have been estimated using optimization algorithms in recent years. The deterministic techniques include Lambert W-functions, least squares, and curve fitting. Because of the parameters’ differentiability and convexity, deterministic methods have several restrictions. Since these techniques are highly sensitive to the initial solution, they tend to arrive at a local minimum rather than the global optimum [8, 9]. A heuristic method is introduced as an alternative to the deterministic method, and it has been proven that heuristics methods can produce more accurate and robust results [10]. There are a variety of heuristic methods that are based on population data and are derived from nature [11]. The use of heuristics is a common application in engineering whereby the use of these algorithms can be used to solve challenges such as differentiability and convexity without the need to address these specific aspects. Several advantages can be gained from using these heuristic methods when attempting to estimate the parameters of solar PV cells. Over the past few years, various heuristic methods have been developed and utilized for the purpose of enhancing the efficiency of these methods. In addition to particle swarm optimization (PSO), genetic algorithms [12], teaching-learning optimization (TLO) [13], cuckoo search (CS) [14], artificial bee colonies (ABCs) [15], Rao-1 [16], and Jaya algorithm [17], there are other notable algorithms. It has been reported that these methods have been successfully applied in the field in many instances by researchers. Table 1 shows the latest optimization techniques used by various researchers to estimate the unknown parameters of solar PV diode model.

**Table 1:** Optimization method for three and four diode model.

Author	Technique/ Algorithm	Solar Diode PV Model				Year
		Single	Double	Three	Four	
M. Abdel-Basset *et al*. [18]	Improved equilibrium optimizer	✔	✔	✔	-	2020
A. A. Z. Diab *et al*. [19]	Coyote optimization algorithm	✔	✔	✔	-	2020
M. Naeijian *et al*. [20]	Whippy Harris Hawks Optimization Algorithm	✔	✔	✔	-	2021
R. Y. Abdelghany *et al*. [21]	Improved bonobo optimizer	✔	✔	✔	-	2021
W. Zhou *et al*. [22]	Metaphor-free dynamic spherical evolution	✔	✔	✔	-	2021
G. Xiong *et al*. [23]	Gaining–sharing knowledge based algorithm	✔	✔	✔	-	2021
A. Singh *et al*. [24]	Tuna Swarm Optimizer with Newton-Raphson method	-	-	✔	-	2022
C. Kumar *et al*. [25]	Hybrid particle swarm optimization algorithms	✔	✔	✔	-	2022
J. Gupta *et al*. [26]	Hybrid Particle Swarm Optimization and Gravitational Search Algorithm	✔	✔	✔	-	2022
M. Premkumar *et al*. [27]	An enhanced Gradient-based Optimizer	✔	✔	✔	-	2022
M. K. Singla *et al*. [28]	Hybrid algorithm	-	-	✔	-	2021
A.-E. Ramadan *et al*. [29]	Improved grey wolf optimizer	-	-	✔	-	2021
P. He *et al*. [30]	Radial Basis Function Based Meta-Heuristic Algorithms	✔	✔	✔	-	2023
B. Singh *et al*. [31]	Hybrid algorithm	✔	✔	✔	✔	2022

### 1.3 Observation

In spite of their effectiveness and speed, heuristic algorithms have few limitations when compared to traditional techniques. Due to the exclusive searching mechanism of PSO and GA, these methods primarily focus on local minima, resulting in a high probability of premature convergence for multi-modal systems. While CS and ABC are effective when exploring at the initial exploration stage, they tend to be slow to achieve convergence once the exploration stage is complete. The result is that when these methods are applied to multi-objective functions, they tend to perform poorly as a result. As a result of the fact that the objective function is derived from noisy raw data, most heuristic algorithms do not perform optimally when applied to noisy raw data. A heuristic approach was needed to address the problem of estimating the parameters of solar PV cells, which aimed to strike a balance between local and global search capabilities using a heuristic method [32]. It has been found that a single diode model incorporating a series resistance can be enhanced in terms of efficiency by adding a series resistance. In order to implement the proposed algorithm, we will take advantage of both three diode and four diode models of solar PV cells, thereby utilizing a new algorithm for parameter estimation. The motivation of the manuscript is to develop a metaheuristic algorithm is can estimate the unknown parameters accurately and can used to sole more complex mathematical model with ease and accuracy. The new algorithm consists of hybridization of PSO as well as RSA algorithm which eliminates the drawback of struck in local minima, therefore estimating the precise value of unknown parameters.

There are a number of major contributions made in this paper, which can be summed up as follows:

- The hybrid (PSORSA) algorithm is justified through five benchmark CEC2019 test functions, and the average and Standard Deviation (SD) are calculated for each of them.
- Solar PV cell parameters are estimated at standard temperature condition and Root Mean Square Error (RMSE) is compared with other standalone algorithms.
- The non-parametric test is performed i.e., Friedman Ranking Test and Wilcoxon’s Rank Sum Test.

## 2. Solar PV cell model mathematical modelling and problem formulation

In order to model a PV cell, there are two primary steps that need to be followed. The first step in the research process is to formulate the mathematical model that represents the PV cell. In the subsequent step, parameters are estimated to determine the specific values for the model’s parameters. Single diode (SD) and double diode (DD) models are among the most frequently explored and reported PV models. To understand and analyze the behavior of solar PV cells, these models serve as fundamental building blocks.

### 2.1 Solar PV cell mathematical modelling

#### Enhancing parameter extraction: A comprehensive analysis of the three-diode model for PV Cells

**Fig 1:**
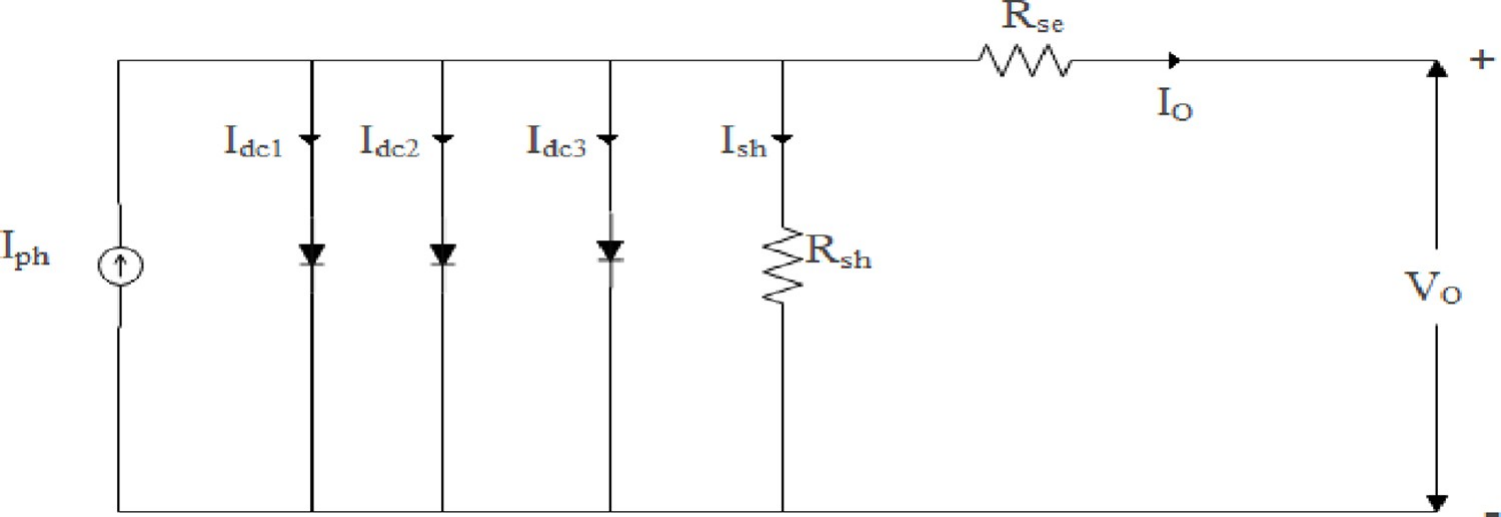
Efficient representation of PV circuit with three diodes.


When it comes to modeling solar PV cells, three diode models offer an improvement over double diode models. In Fig 1, three diodes are illustrated operating within the equivalent circuit model with three diodes. I_dc3_ represents leakage currents and grain boundaries, which are taken into consideration in this model. Using the three-diode equivalent circuit model as an example, leakage current is flowing through the shunt resistance, which is attached to the diode equivalent circuit. In addition, it should be mentioned that the semiconductor-to-substrate resistance in solar PV cells is a representation of the series resistance within the fundamental region. It is through the incorporation of these factors that the three-diode model is able to provide a more accurate and comprehensive representation of the behavior of solar PV cells. Eq 1 illustrates the modelling of three diodes.

$$I_{O}=I_{ph}-I_{rsd1}\left\lfloor exp(\frac{q(V_{O}+I_{O}R_{se}}{n_{1}KT})-1\right\rfloor -I_{rsd2}\left\lfloor exp(\frac{q(V_{O}+I_{O}R_{se}}{n_{2}KT})-1\right\rfloor -I_{rsd3}\left\lfloor exp(\frac{q(V_{O}+I_{O}R_{se}}{n_{3}KT})-1\right\rfloor -\frac{V_{O}+I_{O}R_{se}}{R_{sh}}$$ (1)

The curve fitting accuracy of a three-diode solar PV cell is high, and it is possible to determine the different components of the solar PV cell’s current. However, its modeling is extremely complex. The I-V characteristics of silicon solar cells of large area are simulated using this model.

#### Enhancing parameter extraction: A comprehensive analysis of the four-diode model for PV Cells

**Fig 2:**
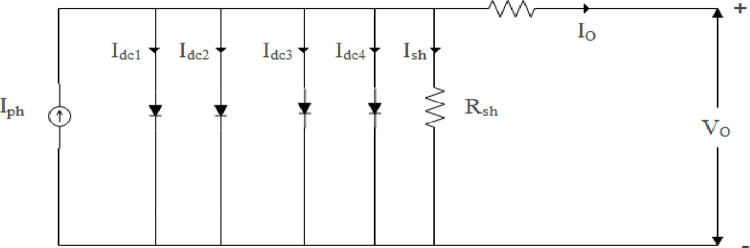
Efficient representation of PV circuit with four diodes.

There are several advantages of using four diode equivalent circuits for the analysis of industrial solar PV cells over single, double, and triple diode models. Compared with other curve fitting algorithms, this software exhibits higher accuracy with minimal error between experiments and calculations, superior curve-fitting capabilities, and excellent performance under the STC algorithm. As a consequence, this model comes with a high level of complexity, which is a notable drawback. There are a number of factors that need to be taken into account when describing the characteristics of solar PV cells in large industrial applications, where PV cell sizes exceed 155.2 cm^2^ and solar PV cell efficiency is 17.1%, and the parameters I_dc1_ and I_dc2_ do not adequately represent solar PV cell characteristics A four-diode equivalent circuit is shown in Fig 2.

Modelling of four- diode model is represented in the Eq 2:

$$I_{O}=I_{ph}-I_{rsd1}\lfloor exp(\frac{q(V_{O}+I_{O}R_{se})}{n_{1}KT}\rfloor -1)-I_{rsd2}\lfloor exp(\frac{q(V_{O}+I_{O}R_{se})}{n_{2}KT}\rfloor -1)-I_{rsd3}\left\lfloor exp(\frac{q(V_{O}+I_{O}R_{se})}{n_{3}KT})-1\right\rfloor -I_{rsd4}\left\lfloor exp(\frac{q(V_{O}+I_{O}R_{se})}{n_{4}KT})-1\right\rfloor -\frac{V_{O}+I_{O}R_{se}}{R_{sh}}$$ (2)

In the following formula, I_o_ is the output current, V_o_ is the output voltage, I_ph_ is the photocurrent. I_rsd1_, I_rsd2_, I_rsd3_, I_rsd4_ represent the reverse saturation currents of the four diodes, and q represents the absolute amount of current flowing through them. Assume that the ideal factors are n_1_, n_2_, n_3_, n_4_; K is the Boltzmann’s constant; and T is the absolute temperature of the P-N junction (in Kelvin).

### 2.2 Problem formulation

An optimization technique helps identify unknown parameters and experimental I-V data for the real system. Based on the four-diode model of solar PV cells, vector defines the solution of the optimization algorithm where $x=[R_{se\;}R_{sh}I_{ph}I_{rsd1}I_{rsd2}I_{rsd3}I_{rsd4}n_{1}n_{2}n_{3}n_{4}]$. In order to minimize the error between measured currents and calculated currents, parameters of solar PV cells are estimated. In homogeneous form, Eqs 1 and 2 can be rewritten as Eqs 3 and 4 to define the objective function, and for the experimental data, the value of can be calculated.


$$f(V_{O},I_{O},x)=I_{O}-I_{ph}+I_{rsd1}\left\lfloor exp(\frac{q(V_{O}+I_{O}R_{se})}{n_{1}KT})-1\right\rfloor +I_{rsd2}\left\lfloor exp(\frac{q(V_{O}+I_{O}R_{se})}{n_{2}KT})-1\right\rfloor +I_{rsd3}\left\lfloor exp(\frac{q(V_{O}+I_{O}R_{se})}{n_{3}KT}\rfloor -1\right\rfloor -\frac{V_{O}+I_{O}R_{se}}{R_{sh}}$$ (3)

$$f(V_{O},I_{O},x)=I_{O}-I_{ph}+I_{rsd1}\left\lfloor exp(\frac{q(V_{O}+I_{O}R_{se})}{n_{1}KT})-1\right\rfloor +I_{rsd2}\left\lfloor exp(\frac{q(V_{O}+I_{O}R_{se})}{n_{2}KT})-1\right\rfloor +I_{rsd3}\left\lfloor exp(\frac{q(V_{O}+I_{O}R_{se})}{n_{3}KT})-1\right\rfloor +I_{rsd4}\left\lfloor exp(\frac{q(V_{O}+I_{O}R_{se})}{n_{4}KT})-1\right\rfloor -\frac{V_{O}+I_{O}R_{se}}{R_{sh}}$$ (4)

For evaluating the difference between measured and calculated currents, Root Mean Square Error (RMSE) is used. RMSE can be calculated using Eq 5.


$$RMSE=\sqrt{\frac{1}{N}\sum_{i=1}^{N}\left(f_{i}(V_{O},I_{O},x)\right)}$$ (5)

This equation is based on an N number of measured data and an x number of solution vectors. To reduce the value of RMSE, parameter estimation of solar PV cells is therefore the key objective.

## 3. Algorithm

A comparison of PSO [33], RSA [34], SCA [35], GWO [36], CSA [37], HBO [38], and PF [39] based approaches demonstrates the superiority of the PSORSA hybrid algorithm. The parameters of polycrystalline PV cells were optimized using three diode and four diode equivalent models. This section briefly discusses hybrid PSORSA.

### 3.1 Enhancing efficiency with the Rat Search Algorithm (RSA)

This study focuses on analyzing the intelligence and social interactions of rats from two different species, and explores the behavioral characteristics of these rats. The different sizes and weights of these rats are examined in order to gain insight into the activities they are involved with within their territorial communities on a daily basis. In addition to grooming, tumbling, hopping, boxing, and chasing, other activities are included in this type of activity. As a result, rats may display violent behavior when competing for prey, leading to the death of some rats during such competitions. The primary objective of this study is to develop a mathematical model of aggressive behavior among rats during chases and battles, which may provide an accurate representation of the behavior of rats during such situations.

#### Mathematical Modeling: Advancing precision in practical applications

*Unraveling the pursuit strategies of living creatures*: *Chasing*. There is evidence that social agonistic behaviors are displayed by animals, such as rats, when they hunt for prey in groups. It is important to note that when we mathematically model this process, we assume that the most effective search agent already knows the location of the prey when the search starts. Upon detecting the movement of the best search agent, the other search agents adjust their positions accordingly. A mathematical Eq 6 is introduced in this study in order to elucidate this mechanism.


$$\vec{Q}=B.\vec{Q_{j}}(z)+D.\left(\vec{Q_{s}}(z)-\vec{Q_{j}}(z)\right)$$ (6)

The position of the rat is represented as $\vec{Q_{j}}(z)$, and the best optimal solution is denoted as $\vec{Q_{s}}(z)$. As a result of Eq 7 and Eq 8, B and D are determined.


$$B=S-z\times \left(\frac{S}{{Max}_{iter}}\right)\;Where,z=0,1,2,\ldots ..,{Max}_{iter}.$$ (7)

D=2.rand() (8)

Random numbers are generated from two parameters, S and D, in the iterative process. Parameter S has values between 0 and 2, while parameter D has values between 1 and 5. B and D play vital roles in enhancing the efficiency of the exploration and exploitation stages by optimizing the performance of these parameters.

*Fighting*: *Understanding the aggressive behavior of organisms*. Rats battle their prey mathematically using Eq 9 to represent the battles they engage in. Rats likely behave aggressively when confronted by their prey during aggressive encounters described by this equation.


$$\vec{Q_{j}}(z+1)=|\vec{Q_{s}}(z)-\vec{Q}|$$ (9)

**Fig 3:**
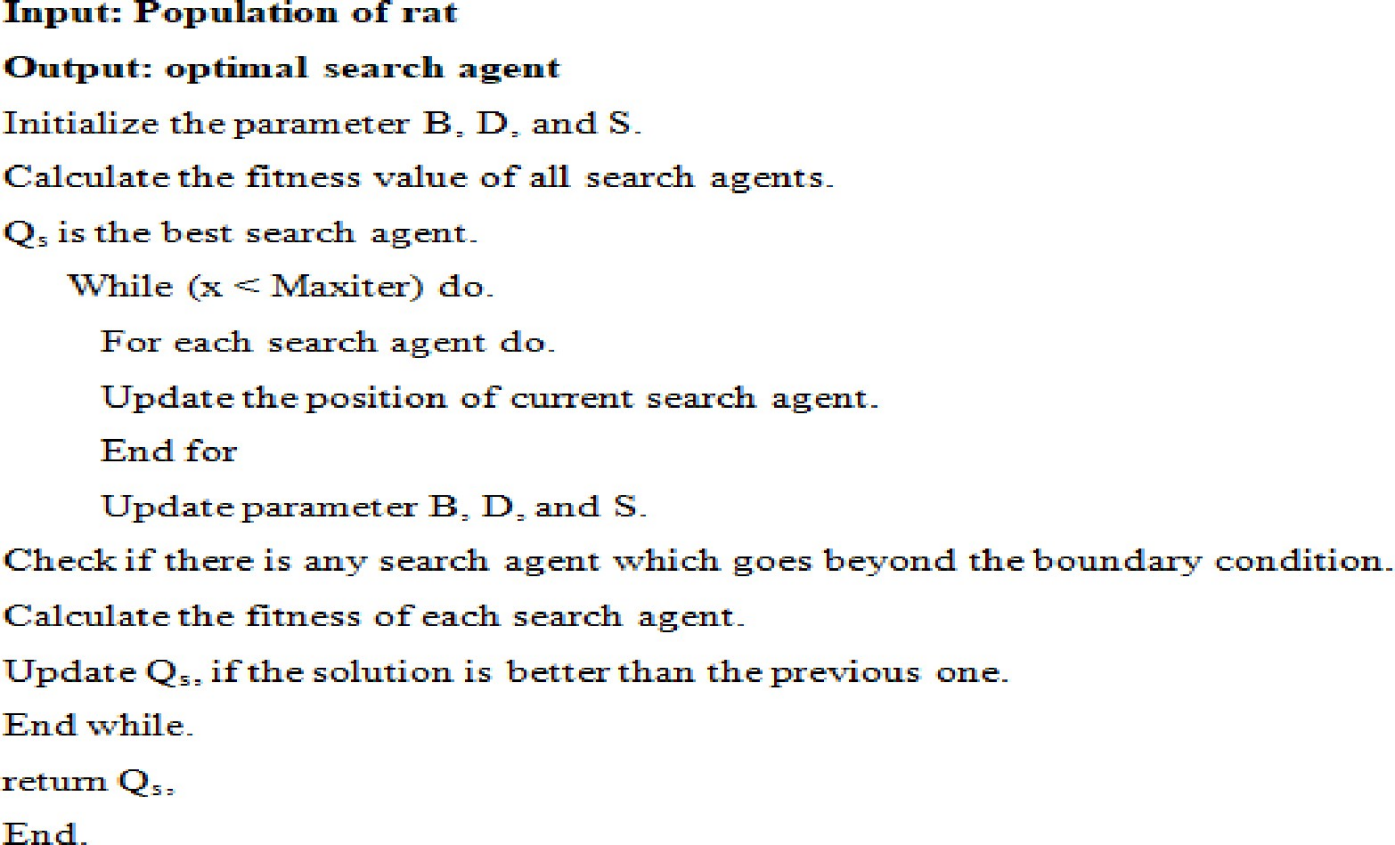
Pseudo-code for an efficient rat search algorithm.

**Fig 4:**
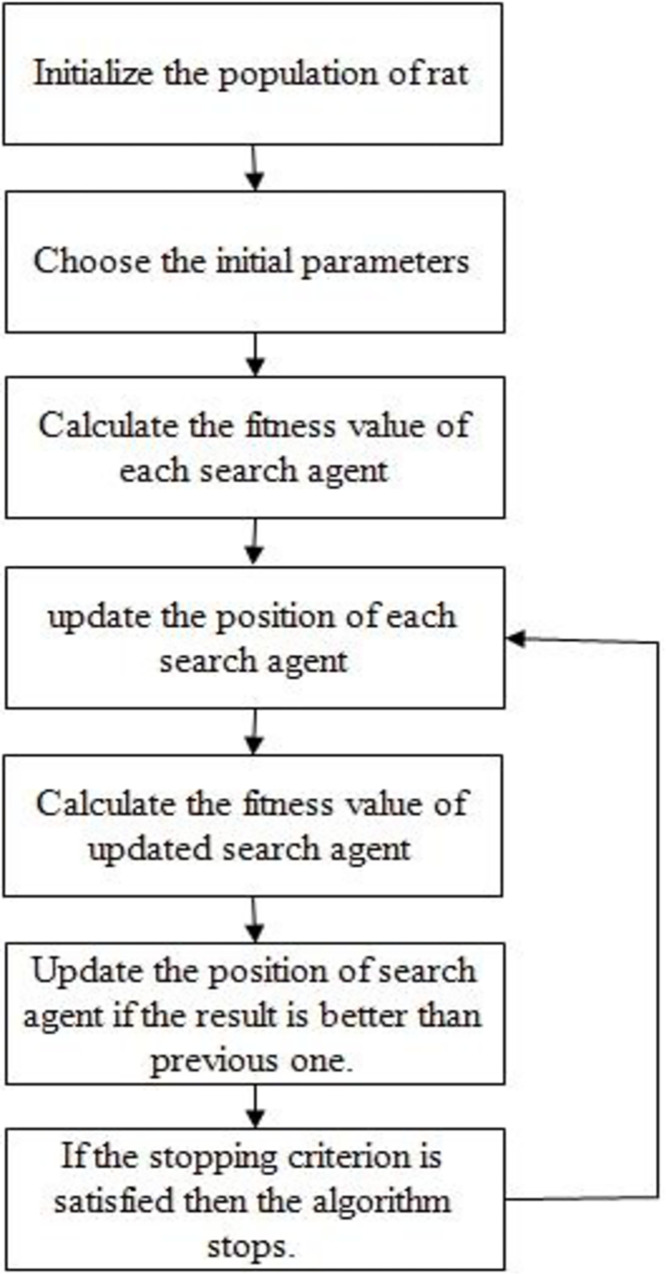
RSA flow chart.

The rat search algorithm employs equation $\vec{Q_{j}}(z+1)$ to update the rat’s next position, ensuring the retention of the optimal solution while adjusting the positions of other search agents accordingly. The parameters B and D are appropriately adjusted to promote effective exploration and exploitation. RSA (Rat Search Algorithm) introduces a solution achievable with a minimal number of operators. Fig 3 presents the pseudo-code for the rat search algorithm, and Fig 4 illustrates the flow chart of the algorithm.

### 3.2 PSORSA: A potent hybrid of particle swarm optimization and rat search algorithm

**Fig 5:**
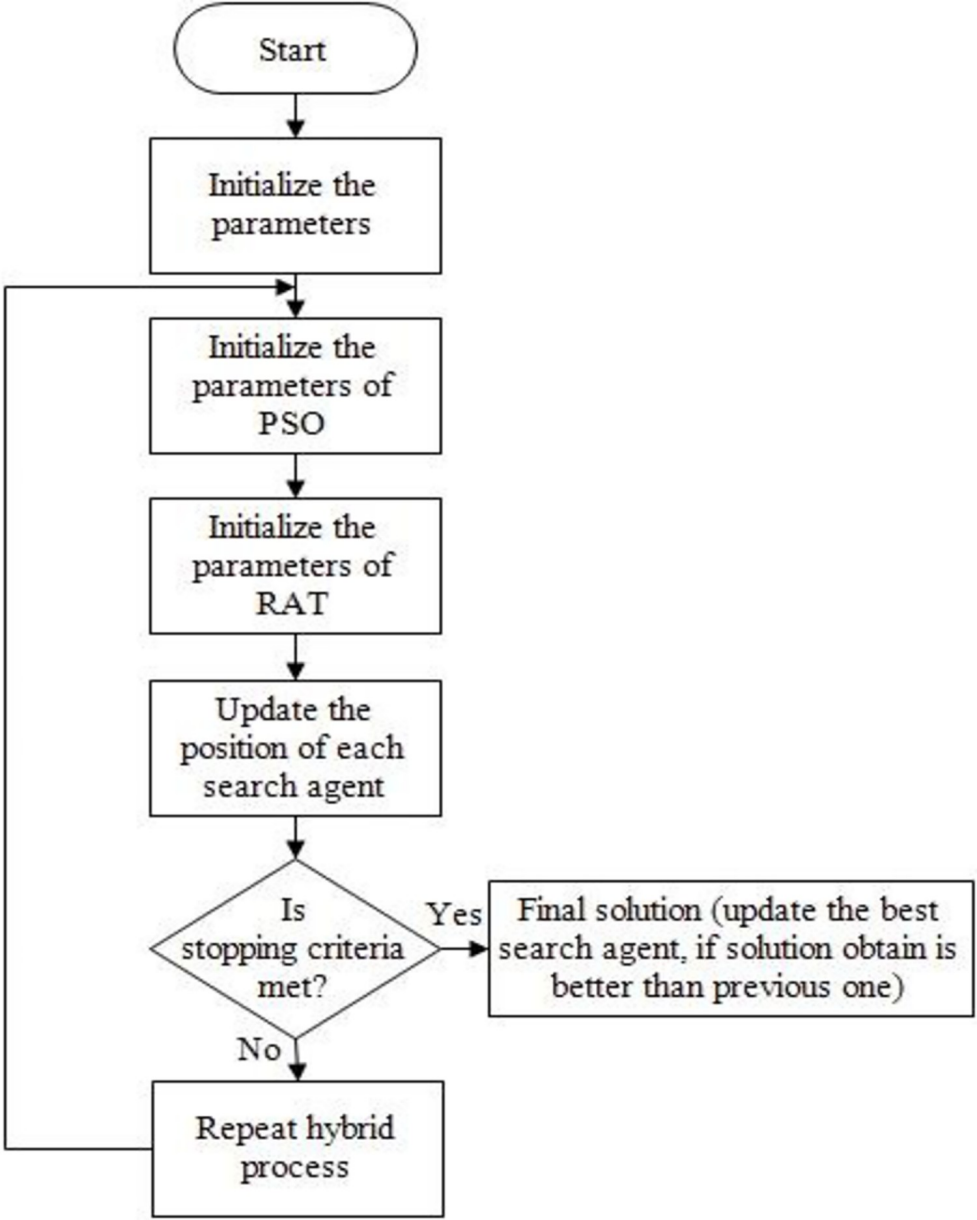
Flow chart of PSORSA.

In this paper, we propose a hybrid approach that combines two algorithms in order to significantly improve efficiency. With the integration of PSO with RSA, a precursor algorithm to swarm algorithms, we improve RSA’s precision. By using the hybrid method, the system is not trapped in local minimums, and it achieves a higher degree of accuracy, resulting in faster operation and the ability to reach the global optimum more quickly. By combining RSA and PSO, we are able to create a powerful hybrid optimization approach. Moreover, as we do not need substantially more computation power to achieve the desired results, our hybrid method proves to be highly cost-effective. For optimizing the system, RSA and PSO can be combined to provide a powerful and cost-effective solution. Fig 5 illustrates the flow chart of this hybrid algorithm.

## 4. Results and discussion

### 4.1 Benchmark test functions

Five benchmark test functions from CEC 2019 are selected to validate the effectiveness of the newly designed algorithm. Table 2 shows the parameters of the algorithm which form the biases for test. As shown in Table 3 these features have dimensions of 20 and 40, with f_1_ to f_5_ being CEC functions. This paper compares PSO, CSA, SCA, RSA, GWO, HBO, PF and PSORSA metaheuristics algorithms to check the performance of proposed algorithms. In order to make a fair comparison between 5 benchmark test functions and other algorithms compared, a limit of 1000 feature evaluations per function is set. Codes were programmed in MATLAB 2018b and each algorithm was run 30 times independently.

**Table 2:** Algorithm parameters.

Algorithm	Parameters	Values
PSO	Search agents	50
	Maximum iteration	1000
	Cognitive factor C1	1.5
	Social factor C2	1.5
	Min inertia weight Wmin	0.4
	Max inertia weight Wmax	0.9
	Random variable r1,r2	Rand [0,1]
CSA	Search agents	50
	Maximum iteration	1000
	Maximum iteration	1000
	Discovery rate	0.24
	cluster	2
SCA	Search agents	50
	Maximum iteration	1000
	a	2
	r1	a-t*((a)/Max_iteration) (decreases linearly from a to 0)
	r2	(2*pi)*rand
	r3	2*rand
	r4	rand
RSA	Search agents	50
	Maximum iteration	1000
	Variable R	[1, 5]
	Variable C	[0, 2]
GWO	Search agents	50
	Maximum iteration	1000
	Random Vector r1, r2	0,1
	Coefficient Vector v	0 to 2
HBO	Search agents	50
	Maximum iteration	1000
	Designed vector C	1
	Parameter $\vec{\lambda }$	2r-1, where r is randon variable [0,1]
PF	Search agents	50
	Maximum iteration	1000
	Npeak	24
	Cmax	50
	Rout/Rcentre	1.5
	Slarge/Ssmall	5
	dred	0.7
PSORAT	Search agents	50
	Maximum iteration	1000
	Cognitive factor C1	1.5
	Social factor C2	1.5
	Min inertia weight Wmin	0.4
	Max inertia weight Wmax	0.9
	Random variable r1,r2	Rand [0,1]
	Variable R	[1, 5]
	Variable C	[0, 2]

**Table 3:** CEC 2019 benchmark test function.

Name of Function	Function	Range
l_1_ = CEC1	f1,f2,f3…f10=Spherefunction δ1,δ2,δ3…δ10=[1,1,1,…1] $\lambda 1,\lambda 2,\lambda 3\ldots \lambda 10=[\frac{5}{100},\frac{5}{100},\frac{5}{100},\ldots \frac{5}{100}]$	[–5, 5]
l_2_ = CEC2	$f1,f2,f3\ldots f10=Griewanks^{\prime }s\;function$ δ1,δ2,δ3…δ10=[1,1,1,…1] $\lambda 1,\lambda 2,\lambda 3\ldots \lambda 10=[\frac{5}{100},\frac{5}{100},\frac{5}{100},\ldots \frac{5}{100}]$	[–5,5]
l_3_ = CEC3	$f1,f2,f3\ldots f10=Griewanks^{\prime }s\;function$ δ1,δ2,δ3…δ10=[1,1,1,…1] λ1,λ2,λ3…λ10=[1,1,1,…1]	[–5,5]
l_4_ = CEC4	$f1,f2=Ackely^{\prime }s\;function$ $f3,f4=Rastrigin^{\prime }s\;function$ f5,f6=Weierstrassfunction $f7,f8=Griewanks^{\prime }s\;function$ f9,f10=Spherefunction δ1,δ2,δ3…δ10=[1,1,1,…1] $\lambda 1,\lambda 2,\lambda 3\ldots \lambda 10=[\frac{5}{32},\frac{5}{32},1,1,\frac{5}{0.5},\frac{5}{0.5},\frac{5}{100},\frac{5}{100},\frac{5}{100},\frac{5}{100}]$	[–5,5]
l_5_ = CEC5	$f1,f2=Rastrigin^{\prime }s\;function$ f3,f4=Weierstrassfunction $f5,f6=Griewanks^{\prime }s\;function$ $f7,f8=Ackely^{\prime }s\;function$ f9,f10=Spherefunction δ1,δ2,δ3…δ10=[1,1,1,…1] $\lambda 1,\lambda 2,\lambda 3\ldots \lambda 10=[\frac{1}{5},\frac{1}{5},\frac{5}{0.5},\frac{5}{0.5},\frac{5}{100},\frac{5}{100},\frac{5}{32},\frac{5}{32},,\frac{5}{100},\frac{5}{100}]$	[–5,5]

Tables 4 and 5 show the average and standard deviations with dimension of 20 and 40 of five benchmark tests obtained by the algorithms. The results of Tables 4 and 5 indicate that the proposed algorithm is more efficient than the others. Comparing the proposed algorithm with the other algorithms, the proposed algorithm is able to demonstrate a lower mean and standard deviation value across five benchmark test functions when compared with the other algorithms. We can conclude from these results from the benchmark test function that the hybrid algorithm that has been proposed outperforms the existing algorithms in terms of convergence rate, robustness, precision, and overall performance as compared to the other algorithms. Therefore, the hybrid algorithm offers better results than the rest and should be used for optimization purposes. It is a reliable and efficient algorithm for solving complex problems.

**Table 4:** CEC 2019 benchmark statistical test with dimension (20).

Algorithms	Functions	l_1_	l_2_	l_3_	l_4_	l_5_
**PSO**	**Average**	1.85E+01	2.20E-01	3.09E-01	3.02E+01	2.96E-01
	**Standard Deviation**	1.97E+01	5.92E-03	7.94E-02	2.20E+01	6.95E-02
**CSA**	**Average**	2.33E+03	2.20E-06	2.39E-06	1.95E+03	1.49E-06
	**Standard Deviation**	1.05E+03	2.93E-06	1.39E-06	9.24E+02	1.32E-06
**SCA**	**Average**	2.84E+01	1.27E-03	1.58E-03	1.08E+01	1.04E-03
	**Standard Deviation**	1.73E+01	9.72E-05	6.25E-04	1.18E+01	4.50E-04
**RSA**	**Average**	2.18E+04	6.13E-13	6.20E-13	2.33E+04	7.82E-13
	**Standard Deviation**	7.69E+03	1.24E-13	8.20E-14	4.03E+03	5.68E-14
**GWO**	**Average**	4.36E+02	3.77E-05	4.66E-05	3.89E+03	5.27E-05
	**Standard Deviation**	2.90E+02	3.05E-05	3.61E-05	3.51E+03	2.47E-05
**HBO**	**Average**	2.84E+03	3.42E-10	1.94E-05	2.15E+03	6.34E-10
	**Standard Deviation**	7.69E+03	2.01E-11	1.20E-05	3.32E+03	3.86E-10
**PF**	**Average**	2.19E+04	4.31E-10	5.86E-12	1.03E+03	6.19E-11
	**Standard Deviation**	2.01E+03	4.63E-10	9.54E-12	1.10E+03	1.01E-12
**PSORSA**	**Average**	2.17E+06	8.37E-17	7.88E-17	2.34E+06	8.56E-17
	**Standard Deviation**	7.48E+05	4.79E-18	6.69E-18	3.00E+05	3.50E-18

**Table 5:** CEC 2019 benchmark statistical test with dimension (40).

Algorithms	Functions	l_1_	l_2_	l_3_	l_4_	l_5_
**PSO**	**Average**	4.27E+01	1.66E-01	1.54E-01	2.66E+01	2.68E-01
	**Standard Deviation**	4.13E+01	3.75E-02	7.62E-02	4.16E+01	9.15E-02
**CSA**	**Average**	3.18E+03	9.61E-07	2.39E-06	3.72E+03	1.28E-06
	**Standard Deviation**	1.42E+03	3.03E-07	1.39E-06	2.37E+03	5.17E-07
**SCA**	**Average**	1.50E+01	2.80E-03	1.15E-03	1.73E+01	8.70E-04
	**Standard Deviation**	8.46E+00	2.81E-03	3.51E-04	1.43E+01	5.42E-04
**RSA**	**Average**	2.27E+04	6.18E-13	5.09E-13	2.07E+04	5.41E-13
	**Standard Deviation**	8.97E+03	5.64E-14	7.49E-14	1.14E+04	3.54E-13
**GWO**	**Average**	2.32E+03	1.72E-05	5.71E-05	3.94E+03	5.85E-05
	**Standard Deviation**	3.05E+03	2.17E-06	2.81E-05	1.25E+03	2.72E-05
**HBO**	**Average**	5.96E+04	1.03E-11	6.16E-11	1.86E+03	2.75E-05
	**Standard Deviation**	9.52E+04	4.23E-11	2.35E-10	1.49E+02	2.13E-05
**PF**	**Average**	3.33E+03	1.38E-08	7.49E-12	4.69E+03	9.44E-12
	**Standard Deviation**	2.92E+03	4.56E-09	5.95E-12	1.80E+03	6.50E-12
**PSORSA**	**Average**	5.00E+06	8.64E-17	8.06E-17	2.25E+06	7.78E-17
	**Standard Deviation**	2.88E+06	3.02E-18	7.09E-18	9.07E+05	1.42E-17

### 4.2 Engineer problem (Parameter extraction of solar cells)

In this section, proposed algorithm addresses parameter extraction issues for two distinct solar PV models to facilitate a deeper analysis of their performance. The specifications of two solar panels from different companies, namely Nemy and Solar World. The first panel is the JP270M60 model from Nemy, which uses mono-crystalline cells. It has a Vm (voltage at maximum power) of 31.10V, Im (current at maximum power) of 8.68A, Voc (open circuit voltage) of 38.60V, and Isc (short circuit current) of 9.20A. The panel consists of 60 cells and is rated for a temperature of 25°C. The second panel is the SW80RNA model from Solar World, which uses poly-crystalline cells. It has a Vm of 17.90V, Im of 4.49A, Voc of 21.90V, and Isc of 4.78A. Like the Nemy panel, it also consists of 60 cells and is rated for a temperature of 25°C. From these two solar panels the parameter extraction of three and four diode model is done. The first parameter is Ipv, which has a lower bound of 0 and an upper bound of 1 (measured in Amperes). The next four parameters, namely Irsd1, Irsd2, Irsd3, and Irsd4, are measured in microamperes (μA) and have a lower bound of 0 and an upper bound of 1. The parameter Rse, measured in Ohms (Ω), has a lower bound of 0 and an upper bound of 0.5. The parameter Rsh, also measured in Ohms (Ω), has a lower bound of 0 and an upper bound of 100. The last set of parameters, n_1_, n_2_, n_3_, and n_4_, are unit less and have a lower bound of 1 and an upper bound of 2. In this section two cases are discussed first one is mono-crystalline and poly-crystalline solar panels.

#### Case 1: Mono-crystalline solar panel

**Fig 6:**
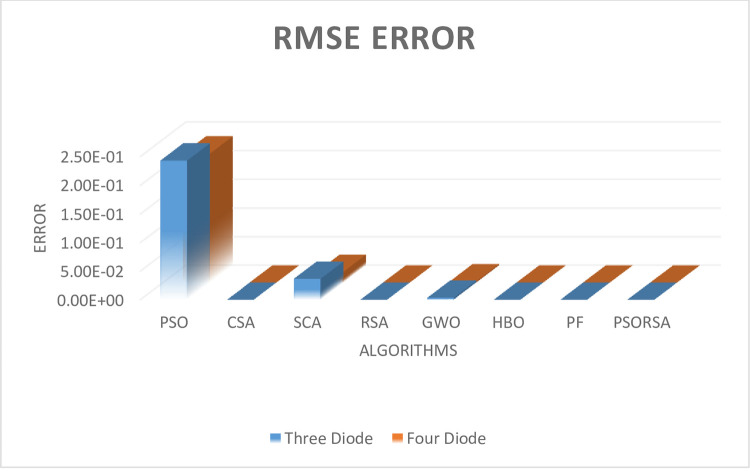
RMSE of both models.

In this case the solar panel is considered as Nemy which is mono-crystalline. In this the parameter extraction of three diode and four diode model is presented. Tables 6 and 7 represents the unknown parameter of both the solar models with respective to error (RMSE). The Fig 6 represents the RMSE error of both the models. From these both table and figure it is concluded that the proposed hybrid algorithm is far better than the compared algorithm. The hybrid algorithm is far better than the standalone algorithm with respect to convergence time, reliability, and memory etc. After the extraction of both the models the Friedman ranking test [40–44] Table 8 and Wilcoxon’s rank sum test [45–51] Table 9 is performed and from this test also it is concluded that the proposed hybrid algorithm is far better than the rest of the compared standalone algorithms.

**Table 6:** Unknown parameters of three diode model.

Parameter/Algorithms	I_pv_	n_1_	n_2_	n_3_	R_s_	R_sh_	I_o1_	I_o2_	I_o3_	RMSE
**PSO**	9.392	1	1.452	0.750	0.003	141.36	0	4.07E-07	3.67E-09	5.66E-01
**CSA**	9.233	1.787	1.681	1.750	0.045	456.46	7.51E-07	7.86E-07	5.36E-07	7.20E-05
**SCA**	9.303	0.750	1.654	1.303	0.018	147.04	0	6.10E-10	6.31E-07	4.62E-02
**RSA**	9.323	1.984	1.676	1.450	0.003	142.52	3.33E-07	6.66E-07	3.59E-07	3.64E-08
**GWO**	9.253	1.763	1.775	1.475	0.051	176.09	2.78E-07	5.76E-07	3.41E-07	6.55E-03
**HBO**	9.748	1.720	1.693	1.438	0.097	1.658	17.6214	5.55E-07	6.22E-07	4.41E-06
**PF**	9.790	1.823	1.662	1.334	0.015	1.568	52.2348	5.41E-07	3.28E-07	2.89E-07
**PSORSA**	9.229	1.652	1.565	1.578	0.078	326.69	3.75E-07	2.44E-07	3.50E-07	3.21E-11

**Table 7:** Unknown parameters of four diode model.

Parameter/Algorithms	I_pv_	n_1_	n_2_	n_3_	n_4_	R_s_	R_sh_	I_o1_	I_o2_	I_o3_	I_o4_	RMSE
**PSO**	9.524	0.678	1.051	0.587	0.5	0.001	28.59	8.26E-11	6.08E-08	0	4.58E-08	4.97E-01
**CSA**	9.191	1.643	1.654	1.699	1.566	0.017	387.00	2.42E-07	5.61E-07	2.01E-07	5.48E-07	7.27E-05
**SCA**	9.285	1.596	1.352	1.846	1.535	0.054	209.02	5.33E-07	0	8.06E-07	2.60E-07	2.05E-02
**RSA**	9.302	1.653	1.802	1.351	1.566	0.015	140.47	3.80E-07	0	1.00E-06	3.E-07	6.27E-08
**GWO**	9.248	1.698	1.400	1.497	1.596	0.081	215.74	3.13E-07	7.15E-08	3.60E-07	1.37E-07	4.30E-03
**HBO**	9.770	1.403	1.595	1.558	1.520	0.057	148.68	3.65E-07	0	5.94E-07	2.78E-07	5.53E-06
**PF**	9.781	1.3293	1.5465	1.5580	1.4799	0.061	208.03	6.75E-07	4.62E-07	5.67E-07	1.95E-07	4.49E-07
**PSORSA**	9.245	1.520	1.658	1.510	1.395	0.078	188.11	2.98E-07	1.52E-07	2.34E-07	1.52E-07	1.86E-11

**Table 8:** Friedman ranking test of both the models.

Algorithms	Friedman Ranking Test
**PSO**	8
**CSA**	5
**SCA**	7
**RSA**	2
**GWO**	6
**HBO**	4
**PF**	3
**PSORSA**	1

**Table 9:** Wilcoxon’s rank sum test of both the models.

Algorithms	PSO	CSA	SCA	RSA	GWO	HBO	PF
**PSORSA vs. (Three Diode Model)**	9.23E-11	8.01E-11	9.10E-11	7.25E-11	8.17E-11	7.96E-11	7.43E-11
**PSORSA vs. (Four Diode Model)**	9.12E-11	7.95E-11	9.01E-11	7.36E-11	8.05E-11	7.88E-11	7.61E-11

#### Case 2: Poly-crystalline solar panel

**Fig 7:**
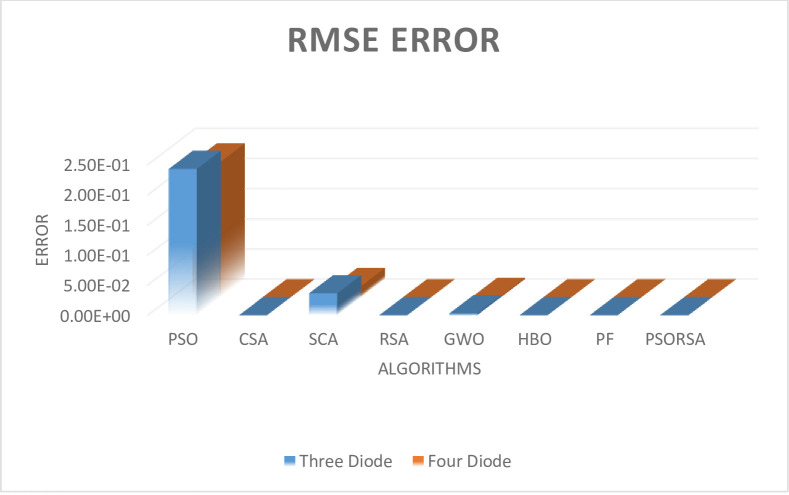
RMSE of both models.

In this case the solar panel is considered as solar world which is poly-crystalline. In this the parameter extraction of three diode and four diode model is presented. Tables 10 and 11 represents the unknown parameter of both the solar models with respective to error (RMSE). The Fig 7 represents the RMSE error of both the models. From these both table and figure it is concluded that the proposed hybrid algorithm is far better than the compared algorithm. The hybrid algorithm is far better than the standalone algorithm with respect to convergence time, reliability, and memory etc. After the extraction of both the models the Friedman ranking test Table 12 and Wilcoxon’s rank sum test Table 13 is performed and from this test also it is concluded that the proposed hybrid algorithm is far better than the rest of the compared standalone algorithms.

**Table 10:** Unknown parameters of three diode model.

Parameter/Algorithms	I_pv_	n_1_	n_2_	n_3_	R_s_	R_sh_	I_o1_	I_o2_	I_o3_	RMSE
**PSO**	4.950	0.5	0.749	0.576	0.001	85.118	0	4.16E-07	7.70E-09	2.43E-01
**CSA**	4.821	1.316	1.094	0.998	0.070	390.68	6.01E-07	4.18E-07	5.08E-07	5.93E-05
**SCA**	4.915	0.714	1.034	0.947	0.017	219.46	3.24E-09	2.66E-07	2.33E-07	3.65E-02
**RSA**	4.850	1.120	1.246	1.557	0.001	159.28	6.66E-07	0	6.66E-07	3.63E-07
**GWO**	4.822	1.316	1.213	1.135	0.096	273.09	7.31E-08	2.78E-07	2.57E-07	3.26E-03
**HBO**	4.872	1.203	1.225	1.558	0.067	235.91	7.68E-08	2.65E-07	6.52E-07	3.96E-06
**PF**	4.815	1.329	1.268	1.558	0.076	258.04	6.80E-07	2.75E-07	6.62E-07	5.65E-06
**PSORSA**	4.858	1.081	1.161	1.261	0.077	158.20	2.69E-07	3.80E-07	6.28E-07	4.14E-09

**Table 11:** Unknown parameters of four diode model.

Parameter/Algorithms	I_pv_	n_1_	n_2_	n_3_	n_4_	R_s_	R_sh_	I_o1_	I_o2_	I_o3_	I_o4_	RMSE
**PSO**	4.800	0.571	0.644	0.5	0.646	0.031	227.47	5.53E-09	3.11E-08	2.07E-09	0	2.25E-01
**CSA**	4.838	1.619	0.956	1.891	1.359	0.038	414.76	3.39E-07	1.42E-07	3.75E-07	5.10E-07	6.50E-05
**SCA**	4.889	1.637	0.972	1.199	0.909	0.027	265.43	2.10E-07	0	5.74E-07	6.60E-07	1.83E-02
**RSA**	4.855	1.366	0.979	1.122	1.044	0.001	134.05	6.66E-07	6.66E-07	6.66E-07	3.33E-07	2.25E-07
**GWO**	4.810	0.940	1.026	1.286	1.068	0.023	301.84	5.24E-07	3.12E-07	3.97E-07	2.17E-08	2.04E-03
**HBO**	4.855	1.362	1.034	1.258	1.053	0.024	247.921	6.32E-07	2.65E-07	6.58E-07	3.88E-07	2.01E-06
**PF**	4.890	1.323	1.062	1.235	1.048	0.025	223.48	5.41E-07	2.28E-06	3.89E-07	3.97E-07	5.34E-07
**PSORSA**	4.828	1.085	1.214	1.089	1.074	0.087	220.96	2.55E-07	4.87E-07	3.74E-07	2.44E-07	4.72E-09

**Table 12:** Friedman ranking test of both the models.

Algorithms	Friedman Ranking Test
**PSO**	8
**CSA**	7
**SCA**	5
**RSA**	2
**GWO**	6
**HBO**	4
**PF**	3
**PSORSA**	1

**Table 13:** Wilcoxon’s rank sum test of both the models.

Algorithms	PSO	CSA	SCA	RSA	GWO	HBO	PF
**PSORSA vs. (Three Diode Model)**	8.54E-09	7.20E-09	8.14E-09	7.02E-09	7.89E-09	7.77E-09	7.54E-09
**PSORSA vs. (Four Diode Model)**	8.24E-09	7.04E-09	8.02E-09	6.89E-09	7.54E-09	6.99E-09	6.91E-09

## 5. Conclusion

The goal of this research is to introduce a novel hybrid algorithm, PSORSA, which will assist in addressing global optimization challenges and extracting solar cell parameters under varying temperatures. PSORSA is a meta-heuristic algorithm that merges two meta-heuristic algorithms, PSO and RSA, to strike a balance between exploring new ideas and exploitation existing ideas. The approach incorporates the opposition-based learning approach, which can be used to enhance the diversity of demographic groups. In order to conduct this study, two solar PV cell models, namely Nemy-JP270M60 and Solar World-SW80RNA, are used, which are mathematically equivalent models of PV cells with three and four diodes. Following are some of the results that have been obtained as a result of this investigation:

- It has been shown that PSORSA is far superior to other algorithms when it comes to achieving precise solutions and achieving faster convergence when it comes to global optimization problems.
- It can be concluded that PSORSA performs better than any of the other techniques in terms of consistency in solutions, as well as equivalent efficiency, when compared to Friedman ranking and Wilcoxon’s rank-sum tests.
- Statistically, both PV models are more effectively managed using PSORSA than they are when extracting parameters for them via a regression analysis.

There is no doubt that the findings of the study establish PSORSA as an effective and promising method for extracting solar PV cell parameters. It is important to note that PSORSA can be applied to a variety of other energy optimization challenges, in addition to solar PV cell parameters, making it an ideal tool for tackling others. The system could be used in the power system arena to address issues such as optimal distributed generation configuration, economic load dispatch, and energy scheduling problems, potentially providing greater chances of achieving successful outcomes in those areas.

## Supporting information

S1 Data (XLSX)
